# Isis Revelata: An Inquiry into the Origin, Progress, and Present State of Animal Magnetism

**Published:** 1837-10

**Authors:** 


### Art. VIII.

*Isis Revelata: an Inquiry into the Origin, Progress, and present State of Animal Magnetism.* By J. C. Colquhoun, Esq., Advocate, F.R.S.E. —*Edinburgh*, 1836. Two Vols. 8vo. pp. 395, 416.

It was our intention to take some notice of Mr. Colquhoun's strangely-named book, in one of the articles on the Physiology and Pathology of the Nervous System, given in our last two Numbers, but were prevented by want of room. On the present occasion, we do not propose to give any minute account of the work before us, nor yet to enter at large upon the consideration of the doctrines upon which it treats; but merely to hazard a few general observations upon Animal Magnetism, and with the hope rather of placing the subject in a proper point of view, than of enabling our readers to arrive at a positive conclusion in regard to it.

That a very peculiar temporary condition of the functions of the nervous system has been frequently observed, and described under the names of Somnambulism, Extase, Clairvoyance, Crisis, Reverie, &c.; that it has been produced in very various ways, and, although presenting certain pretty uniform characters, has varied remarkably in the phenomena it has exhibited in different persons and at different times,—no one who has given any attention to the subject will dispute. The points really in dispute are two: 1. Is it true that any influence can be transmitted from one individual to another, capable of producing this state of somnambulism? 2. How far is the natural condition of the functions of the nervous system, and particularly of the sensations, and of the inferences immediately deduced from the sensations, capable of being altered in such cases?

1. The first question was supposed to have been satisfactorily answered by the committee of the French Academy of Sciences, of which Bailly was reporter and Franklin a member, and which framed the well-known Report published in 1784. The decided opinion of that committee was, that no such influence, capable of transmission from one individual to another, exists; and it appears quite certain that by far the greater number of cases of somnambulism have either occurred spontaneously or been excited by mental emotions, or propagated by irritation; and that many, thought to have been owing to a peculiar transmitted influence, have been really produced in one of the two last-mentioned ways. But all this does not prove the impossibility of such an influence being, in certain circumstances, communicable from one person to another; and, when we remember that Cuvier expressed himself favorably as to the possibility of such an influence; that Andral states his belief that the state of extase "can be produced by an influence exercised by one individual on another," and that "imitation and imagination are inadequate to explain these phenomena;" and when we read the following opinion of the committee of the Royal Academy of Medicine, appointed to investigate this subject in 1826, (bearing in mind, also, the superior importance of a *positive* to a *negative* fact,) we think we are not warranted in adhering *simpliciter* to the judgment of the former committee, and holding the question decided in the negative. The Report of the Committee of the Academy of Medicine, contained in the work before us, is signed by

Bourdois de la Motte, Fouquier, Gueneau de Mussy, Guersent, Husson, Itard, J. J. Le Roux, Marc, Thillaye; and, although we should like to understand distinctly why Messrs. Magendie and Double, although appointed to the committee, did not attend at the experiments, and therefore did not sign the Report, yet we think it must be allowed that several of the names attached to it are deserving of the most respectful consideration. Their opinion as to the question now before us is expressed pretty decidedly.

"We hold it as demonstrated, that the somnambulism has been produced in circumstances in which the persons magnetized could not see, or were ignorant of, the means employed to occasion it. . . We can not only act on the magnetized person, but even place him in a complete state of somnambulism, and bring him out of it, without his knowledge, out of his sight, at a certain distance, and with doors intervening." (*Isis*, vol. ii. p. 286.)

The committee refer to two facts observed by them, in illustration of this position, but of these the latter only seems to us of much value. This is the case of a man who had previously exhibited the phenomenon, but was kept in conversation by the members of the committee for half an hour, without any change taking place in him. "M. Foissac, the magnetiser, remained during this time in an antichamber, separated from the room where the patient was by two closed doors and at a distance of twelve feet." He then began his operations, and, after three minutes, the person acted on said, "I believe M. Foissac is there, for I feel myself stupified." At the end of eight minutes he was completely asleep, and then exhibited several of the phenomena of somnambulism. The same thing happened a second time, in the case of the same individual, (P. 266.) This fact, if standing alone, might not seem of much weight, but it is to be considered as a confirmation of many facts stated by the writers on Animal Magnetism, and which have been regarded by many as unworthy of credit.

2. The more important question, however, is as to the degree of change of the usual functions of the nervous system which may take place during the state of somnambulism. It is quite certain that the faculty of sensation, or perhaps more frequently the faculty of attention to the sensations excited, is very singularly modified. Certain sensations produce their wonted effect, and certain trains of thought succeed them; but many other impressions made on the organs of sense, which, if attended to, would interrupt these trains, are either altogether unfelt, or at least fail to produce their wonted effects, whether mental or bodily.

"The greater number of somnambulists whom we have seen were completely insensible. We might tickle their feet, their nostrils, and the angles of their eyes, with a feather; we might pinch their skin so as to leave a mark, prick them with pins under their nails, &c., without producing any pain, without even their apparently perceiving it. Finally, we saw one who was insensible to one of the most painful operations of surgery, and did not manifest the slightest emotion in her countenance, pulse, or respiration." . . . . "During the greater part of the time, they are completely strangers to any external and unexpected noise which is made close to their ears; such as the sound of a copper vessel struck briskly near them, the fall of a piece of furniture, &c." (Vol. ii. p. 287.)

We have ourselves witnessed, in the cases of spontaneous somnambulism, such a degree of apparent insensibility to external impressions as to have less difficulty in giving credit to the accuracy of the whole of this statement. In some instances it appears, as stated by Andral, that

"The magnetised person is capable of maintaining a certain kind of connexion with the external world, while otherwise completely insensible. Thus, he has been known to have answered connectedly various questions and observations proposed to him by one individual, while he remains insensible to the loudest noises, the most exciting remarks, of all the other persons about him. It is difficult not to admit that this has happened in several cases. I have not seen the fact myself, (he adds,) but I have, in the course of reading, met with several instances of it so well authenticated that I should not be justified in refusing to believe it." (*Isis*, vol. ii. p. 137.)

It is equally certain that, while there is a total failure of certain of the most usual associations of thoughts in the mind, there are, in general, certain trains of thought which the mind readily pursues, and in which it often displays an unwonted and extraordinary energy and acuteness. Lastly, it is quite certain that, in the greater number of these cases, after the fit of somnambulism is over, according to the uniform statement of the patients, there is no recollection of what had happened during it; but that, when another paroxysm occurs, the recollection of what had happened, and of the whole train of thought that had occupied the mind during the former paroxysm, is restored. We have seen cases exemplifying all these points, and we might refer to many perfectly authenticated cases, in the works of Abercrombie, (*On the Intellectual Powers*, 5th Edition, p. 314 *et seq.*;) of Darwin, (*On Reverie*, *Zoonomia*, chap. iii. 1, 2, 3;) and the late Dr. Dyer, (*Edinburgh Medical Transactions*, vol. ix.) &c., as clear evidence that, so far as we have hitherto described the state of somnambulism, or extase, it is of real and not very rare occurrence; sometimes complicated with curious perversions of the function of voluntary motion, sometimes without such complication; and that such modification of the functions of the organs of sense and of the mental constitution may occur without any such change in the nervous system as is incompatible with the complete, and even *instantaneous*, restoration of the healthy condition of all its functions.

But a further question remains, which some of our readers may think us absurdly credulous for entertaining at all, and on which we do not pretend to give a decided opinion,—viz. whether, in some of the individuals in this state, the usual conditions of the senses are altered, or the human mind endowed with powers of acquiring information which are denied to it during its connexion with a nervous system in the natural state?

We pronounce no judgment, as yet, on this question, further than by saying that it is one of the highest possible interest, and which it is incumbent on physiologists fairly and candidly to meet, as opportunities may occur, and decide on the evidence of sense and of testimony, independently of all previous prepossessions or opinions. It is very possible,—perhaps, after all, the most probable supposition,—that, in the cases we are about to quote from Mr. Colquhoun, some undetected fallacy or imposition exists, and that, instead of what he terms a "transference of sensations from their usual and appropriate seat to other parts of the nervous system," the information which was obtained by the persons observed really found its way through the usual channels of communicatian between the sensible world and the sentient soul. All that we contend for is, that, as men of intelligence and integrity, whose scientific character was at stake in the observations they made, could not detect such fallacies,—and as we profess ourselves unable, from reading the histories, to conjecture where they had lain, we stand in need of further observations to determine the question, whether such a modification of the mental powers of sensation and perception as he supposes is really possible.*

The following are extracts from the Report of the Committee of the Academy of Medicine, the members of which have been named above.

"M. Petit was magnetised, in presence of the committee, on the 15th March, 1826, and set asleep in the space of a minute. A candle was constantly held, during the experiments, before the eyes of M. Petit, at a distance of one or two inches, and several persons had their eyes constantly fixed on his. None of us could perceive the slightest separation of the eyelids. M. Ribes, indeed, remarked that their edges were superimposed, so that the eyelashes crossed each other.†

"M. Ribes, member of the Academy, presented a catalogue which he took from his pocket. The somnambulist, after some efforts which seemed to fatigue him, read very distinctly the words, 'Lavater. Il est bien difficile de connaitre les hommes,' &c.'" . . . "A closed letter was presented to him: he could not discover any of its contents, though he followed the direction of the lines with his fingers. But he easily read the address, though it contained a pretty difficult name." . . . "We never ceased to examine the eyes, and to hold a candle near them;" . . "as far as it was possible to judge by the senses, the eyelids were exactly closed."

Again:

"On the 12th January, your committee met at the house of M. Foissac, when there were present M.——, a deputy, M. de ——, aide-de-camp of the king, and M. Segalas, member of the Academy. M. Foissac told us that he was going to set Paul Villagrand asleep; that, in the state of somnambulism, a finger should be applied to each of his closed eyes, and that, in spite of this complete occlusion of the eyelids, he should distinguish the colour of cards, read the title of a work, and words or lines pointed out at random in the body of the work. At the end of two minutes of manipulations, Paul fell asleep. The eyelids being kept closed, constantly and alternately, by MM. Fouquier, Itard, Marc, and the reporter (Husson,) there was presented to him a pack of new cards. . . The cards were shuffled, and he recognized easily and successively the king of spades, ace of clubs, queen of spades, nine of clubs, &c." . . . "While his eyelids were kept closed by M. Segalas, there was presented to him a volume which the reporter had brought with him. He read on the title-page 'Histoire de France.' The book was opened at the 89th page, and he read in the first line 'le nombre de ses,' passed over the word 'troupes,' and continued, 'Au moment ou on le croyait occupé des plaisirs du Carnaval.'" . . . "A piece of paper was presented to him, on which were written the words Agglutination and Magnetisme Animal. He spelt the first, and pronounced the two others, &c." . . . . "In all of these experiments, the fingers were applied to the whole of the commissure of both eyes, by pressing down the upper on the under eyelid; and we remarked that the ball of the eye was in a constant rotatory motion, and seemed directed towards the object presented to his vision." . . . "At another sitting, on the 13th March, Paul attempted in vain to distinguish different cards applied to the pit of his stomach; but he read, with his eyes still closed, in a book opened at random, and at this time it was M. Jules Cloquet who kept his eyes shut." (*Isis*, vol. ii. p. 332 *et seq.*)

* For some explanation of this phenomenon, according to the views of certain German philosophers, by a predominant and independent action of the ganglionic system of nerves, see our Review of Dr. Jahn's work in the last Number, p. 125.—Ed.

† It should be remarked that, during all these experiments, absolute silence was preserved.

The following is a quotation from the work of M. Foissac, entitled "Cures Opereés en France par le Magnetisme Animal;" but it professes to be a *procès verbal*, drawn up, during a sitting on 7th March, 1828, by M. Ribes, Professeur agregé at Montpellier, and signed by that gentleman and nine other medical or scientific men.

"M. Foissac having placed the Sieur Paul in a state of somnambulism, a bandage was applied to the eyes of the latter, and M. Borel, placed behind the somnambulist, pressed his fingers on the lower part of the bandage, in such a way as to interrupt the view between the stomach and eyes. M. Ribes gave him the nine of hearts, which the somnambulist placed opposite the naked epigastric region: after examining it for some minutes, he requested another card. M. Juglar gave him the king of spades, which the somnambulist named, after examining it for some minutes, without exploring it by the touch. M. Ribes then pressed his fingers on the lower part of the bandage, and M. Juglar presented to Paul the queen of hearts, which the latter named after some minutes of attention."

The bandage was then taken off, the eyelids kept closed by the fingers of different persons, as formerly, and a book and different papers, written at the time, presented to him, from which he read various words correctly.

It is to be remembered that these are not isolated cases; they are the results of careful experiments, made to test the accuracy of many previous statements of similar facts, in the same and other persons.

We add only the following statement from the American Journal of the Medical Sciences, (August, 1834.) We happen to know that this case (that of Jane Rider,) was carefully considered and repeatedly examined by many most intelligent and respectable men, and that the truth of the statement, made by Dr. Belden, seemed manifest to them.

"On the 20th November, the reporter took a large black silk handkerchief, placed between the folds two pieces of cotton batting, and applied it (to the somnambulist, during the paroxysm,) in such a way that the cotton came directly over the eyes, and completely filled the cavity on each side of the nose; the silk was distinctly seen to be in close contact with the skin. Various names were then written on cards, both of persons with whom she was acquainted and of others unknown to her, which she read as soon as they were presented to her. . . . Being desirous, if possible, to prove that the eye was actually closed, the reporter took two large wads of cotton, and placed them directly on the closed eyelids, and then bound them on with the handkerchief before used. The cotton filled the cavity under the eyebrow, came down to the middle of the cheek, and was in close contact with the nose. The former experiments were then repeated, without any difference in the result." (*Isis*, i. 377-8.)

The only explanation that suggested itself to the gentlemen who examined this case was, that the sensibility of the eyes during the paroxysms was such, that the rays of light which penetrated these bandages were sufficient to form images on the retinæ adequate for distinct vision; a supposition which is obviously inapplicable to many of the alleged cases of apparent transference of sensation to the epigastrium and other parts of the body.

We have said that we are aware many of our readers will think us absurdly credulous for dwelling at all on such cases; but, when the evidence of testimony is so strong, we think we may justly take the liberty of enquiring into the grounds of this determined scepticism. Now, the main reason of it (and certainly a very sufficient ground for incredulity and caution,) is, that, if these statements are true, not only the endowments of certain nerves, but the *conditions of certain senses*, must have undergone a change. Those articles of knowledge which, in the natural state, we acquire only by means of inverted images on the retina of the eye, formed by rays of light passing off from external objects, and refracted by the transparent parts of the eye, would here seem to be communicated to the human mind in circumstances where these conditions cannot be fulfilled. This is undoubtedly *inexplicable* and improbable; but, before we assert that it is *impossible*, we ought to ask ourselves, Is vision by means of the inverted images on the retina explicable? Can we explain how it happens that, in consequence of images of certain forms and sizes being formed there, we acquire the knowledge of external objects of particular figures, (often very different from those of the images in question,) being placed at particular distances, and in particular directions, from our eyes? If we can *explain* how these things are taught us by the inverted images on our retinæ, or deduce those articles of knowledge, by logical reasoning, from the sensations which these images necessarily excite, then we may be entitled to say that vision, without these preliminary conditions, is impossible. But *if vision, by means of images on the retinæ, is truly inexplicable*,—if it is truly a mystery how we get the information which these images communicate to us,—how are we entitled to say that vision may not take place without these images; i.e. that the same information may not be communicated to us by some other means?

Now it is quaintly, but we believe quite justly, observed by the late Dr. Brown, that "vision is, in fact, the art of seeing things which are invisible;" i. e. of acquiring information, by means of the eye, which is neither contained in the sensations of sight themselves, nor logically deducible from the intimations which those sensations really convey. If, indeed, the acquisition of knowledge by the eye were known to be truly an *art*,—i. e. if it were only by experience and association with the sense of *touch* that we learn how to obtain information as to the distances, forms, and magnitude of visible objects, (as Dr. Brown and many others since the time of Berkeley have supposed,) we might justly question the possibility of these acquired perceptions of sight being made known to us, in any way, without a similar process of education being undergone. But we distrust part of that theory, even in relation to the human species; and Dr. Brown himself abandons it in regard to the lower animals, admitting that they are endowed by nature with the power of judging of the size, form, and distance of objects placed before their eyes, prior to all experience, and notwithstanding that these particulars are not truly objects of sight.

Even in the case of man, we apprehend that any one who has studied with attention Dr. Reid's argument on Perception, as distinguished from Sensation, on the utter discrepancy between the notions that our minds are so constituted as to form of the primary qualities of matter, and the sensations by which these notions are suggested to our minds, (and any one who has not studied that argument is, we conceive, very incompetent to judge of this question,) must admit that the same principle applies at least to certain of our sensations, and by the inferences from them. The very notion of external and independent existence, essential to our conceptions of all external objects, the notions of extension and of hardness, are so perfectly distinct from the sensations which naturally excite them in the mind, that they all exemplify what D'Alembert called the "*instinct prior to reason, and surer than reason,* by which we shoot the gulf which separates the sensible world from the sentient soul." In other words, we must admit the principle of *intuition* as one source of the information which we, as well as other animals, derive from the senses. Now, if we admit this principle, how are we to assign limits to its operation? If it is only by this principle of intuition, which is *truly inexplicable,* that we, or other animals, acquire part of the information from the inverted images on the retinæ which they are capable of affording us, how can we judge, otherwise than by experience, whether or not circumstances may exist under which the same information may be communicated to our minds, without the intervention of any inverted images?

This we apprehend to be the strictly philosophical point of view in which these alleged instances of transference of sensations from one part of the body to another (or, more correctly, of alteration of the conditions under which the mind acquires information by the exercise of the senses,) should be considered; and if further and more decisive observations shall determine this question in the affirmative, we would regard such facts as striking and important illustrations of the truth of the principle which has been maintained by Reid and his followers,—viz. that an essential part of the knowledge which we acquire during the exercise of our senses is to be ascribed only to the constitution of our minds, leading us, *intuitively and irresistibly,* to form certain general notions, and deduce certain inferences from our sensations, which are neither contained in nor logically deducible from these sensations themselves.

If it be indeed true that, under any circumstances, the human mind can dispense with the ordinary ministration of the senses, and acquire such information as it is their office to afford, unfettered by the conditions which they impose, the circle of human science will hardly present a truth of equal interest and importance. That facts should have been observed of weight sufficient to induce many men of undoubted veracity and of high scientific acquirements to believe this truth established, is, of itself, one of the most striking events of the present age. A sound logic, as we firmly believe, is not only sufficient to establish, but demands the recognition of the immutable distinction of mind and matter, independently of any such deviation from the ordinary laws of nature; but we are not surprised that the conviction of having actually witnessed such a deviation should have impressed the mind of one previously bewildered by sophistry on this subject, in the manner which is strikingly represented in the dying declaration of M. Georget: "In my Physiology of the Nervous System I boldly professed materialism; but, scarcely was it given to the world, when new meditations on the extraordinary phenomenon, somnambulism, no longer permitted me to doubt of the existence in us, and without us, of an intelligent principle, altogether different from material existences. Let it be, if you will, the soul, or God. I have, in regard to this, a profound conviction, founded on facts which I believe to be incontestable. This declaration will not see the light until there can be no longer any doubt of my sincerity, or any suspicion of my intentions."

We have left ourselves no room to consider the other alleged faculties of certain somnambulists,—their alleged powers of perception at incredible distances, and of divination and prophecy. In so far as the prophecies relate to their own health, they are obviously very liable to suspicion; and, in so far as they relate to the actions of others, we think the evidence generally unsatisfactory; and, on the whole, the pretension to these powers seems to us to weaken rather than strengthen the claims of the narratives before us on our belief. We shall only further add, that it is as physiologists and pathologists that we regard the subjects of somnambulism and animal magnetism as of the highest interest and importance. The state of the living body thus designated is decidedly and seriously morbid; and, although we do not doubt that, in certain states of the system, it may be excited with good effect, we cannot suppose that any rational physician, who knows what are the physical evils that most afflict humanity, and "what can be done and what cannot be done" by his art, can ever expect the artificial excitement of it to be safely and beneficially employed, as a remedial agent, on more than a very small number of cases of disease.

